# Functional Analysis of Peptidyl-prolyl *cis-trans* Isomerase from *Aspergillus flavus*

**DOI:** 10.3390/ijms20092206

**Published:** 2019-05-05

**Authors:** Saleem Ahmad, Sen Wang, Weizhong Wu, Kunlong Yang, YanFeng Zhang, Elisabeth Tumukunde, Shihua Wang, Yu Wang

**Affiliations:** 1 Key Laboratory of Pathogenic Fungi and Mycotoxins of Fujian Province, Key Laboratory of Chemical Biology and Biopesticide of Education Ministry, and School of Life Science, Fujian Agriculture and Forestry University, Fuzhou 350002, China; ahmad.chilas@gmail.com (S.A.); zxl521ws@gmail.com (S.W.); wwz1211@163.com (W.W.); ykl_long@yeah.net (K.Y.); zhangy11@gmail.com (Y.Z.); elitumu09@yahoo.fr (E.T.); 2 College of Life Science, Jiangsu Normal University, Xuzhou 221116, Jiangsu Province, China

**Keywords:** *A. flavus*, PPIase, Purification, Enzyme Activity, Protein Model, pathogenicity

## Abstract

*Aspergillus flavus*, a ubiquitous filamentous fungus found in soil, plants and other substrates has been reported not only as a pathogen for plants, but also a carcinogen producing fungus for human. Peptidyl-Prolyl Isomerase (PPIases) plays an important role in cell process such as protein secretion cell cycle control and RNA processing. However, the function of PPIase has not yet been identified in *A. flavus.* In this study, the PPIases gene from *A. flavus* named *ppci1* was cloned into expression vector and the protein was expressed in prokaryotic expression system. Activity of recombinant ppci1 protein was particularly inhibited by FK506, CsA and rapamycin. 3D-Homology model of ppci1 has been constructed with the template, based on 59.7% amino acid similarity. The homologous recombination method was used to construct the single *ppci1* gene deletion strain Δ*ppci1*. We found that, the *ppci1* gene plays important roles in *A. flavus* growth, conidiation, and sclerotia formation, all of which showed reduction in Δppci1 and increased in conidiation compared with the wild-type and complementary strains in *A. flavus.* Furthermore, aflatoxin and peanut seeds infection assays indicated that *ppci1* contributes to virulence of *A. flavus.* Furthermore, we evaluated the effect of PPIase inhibitors on *A. flavus* growth, whereby these were used to treat wild-type strains. We found that the growths were inhibited under every inhibitor. All, these results may provide valuable information for designing inhibitors in the controlling infections of *A. flavus*.

## 1. Introduction

*Aspergillus flavus* is ubiquitous specie of filamentous fungi, which is found widespread in soil, plants and other variety of substrates. *A. flavus* was reported as a pathogen for plants in 1920 [1,2]. This notorious fungus is second to *A. fumigatus* that causes a series of invasive diseases known as aspergillosis in human [3,4]. *A. flavus* produces toxic secondary metabolites known as aflatoxin (AF), which are considered as strong carcinogens [5,6,7], also cause disease in essential agriculture crops, such as maize, wheat and some oil seeds [8]. Therefore, to understand the development of novel strategies against pathogenicity, it is important to investigate the therapeutic targets, and molecular mechanisms of inhibition may enable to control the infections caused by *A. flavus*.

The peptidyl *cis-trans* prolyl isomerase (PPIases) was first isolated by Fischer in 1984 [9]. Which is found in both prokaryotes and eukaryotes [10]. PPIases are enzymes that have catalytic activity for *cis-trans* isomerization at the *N*-terminus site of proline residues. *Cis* to *trans* conformational change of the peptide bond is necessary during protein folding [11,12,13]. The change at thermal equilibrium depends on the different free energy (ΔG) at *cis* or *trans* status [14]. PPIases are unique in their functionality**,** have the ability to keep stabilize *cis-trans* position by lowering the activation energy of products and speed up the isomerization [15,16]. Furthermore, they play important roles in the transportation of Ca^2+^ and several different ions [17]. PPIases also participate in the cell process, such as signal transduction, cell cycle control, growth regulation, protein secretion, apoptosis, RNA processing, association host-pathogen and photosynthesis [18]. Moreover, protein from *Aspergillus nidulans* has been studied more recently in the phytopathogenic field [10]. Members of this family play an important role in morphogenesis and pathogenicity of fungus, such as *Magnaporthe oryzae* [19], *Neurospora crassa* [20], and *Cryphonectria parasitica* [21]. PPIases have been classified as immunophilins by their affinity for immunosuppressive ligands FK506 and cyclosporin A (CsA) [22,23]. FK506 is a fungal polyketide synthesized by *Streptomyces tsukubasesis* which was described as a potent immunosuppressant [24]. FKBP12 was shown to possess PPIase activity, inheritable upon binding to FK506 and rapamycin [25].

There are variety of PPIases that have been reported with different names by their molecular weights, species names and types [23]. Numerous studies have been reported the deletion mutants which show very subtle phenotypic changes under laboratory conditions [26]. Many in vitro or in vivo observable phenotypes of mutants and interactions of PPIase-proteins which seem to be independent of the enzymatic property [27]. In many instances, deletion of the PPIase domain or diminishment of its activity by amino acid substitutions had small impact on protein-protein interactions as well as chaperoning activities [28].

The study of PPIase in *A. flavus* has not been conducted both in vitro and in vivo. Therefore, in this study, the gene (AFLA_0507601) from *A. flavus* (NRRL3357) was cloned by a PCR (Polymerase chain reaction) method and the target gene named as *“ppci1*” was heterologously expressed with an *E. coil* expression system. Then, purification, identification and enzyme activity of the *ppci1* product were analyzed. To know the role of *ppci1* in *A. flavus* in vivo, the homologous recombination method was used to construct the *ppci1* gene deletion mutant Δ*ppci1*. The result showed that *ppci1* played important roles in growth, asexual development and aflatoxin production, sclerotia formation and pathogenicity. All these results display new insights into the role of *ppci1* in *A. flavus* on the basis of prevention and control of *A. flavus* pathogenicity in earlier stages, and guides understanding of the regulation in other pathogenic fungi. This study also provides a novel approach for new promising control strategies for this fungal pathogen, as this gene and the resulting protein may be a crucial target for designing the antifungal drugs.

## 2. Results 

### 2.1. Bioinformatics Analysis of the Sequences

**Figure 1:**
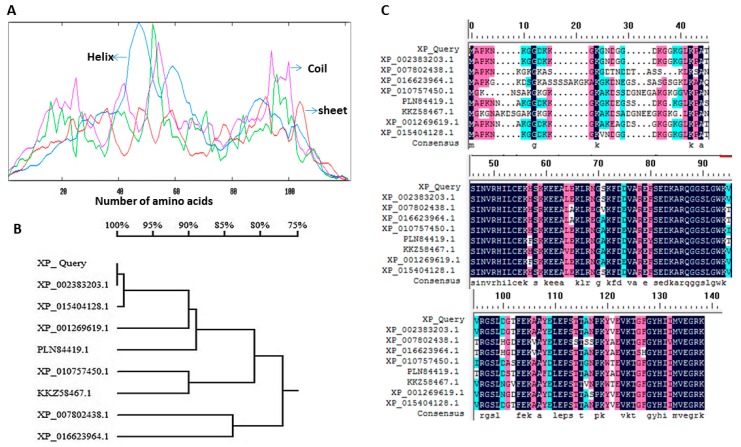
Sequence and phylogenetic analysis. (**A**) Distribution of secondary structure elements in ppci1. Red color shows sheet, purple for coil and blue for the helix. The *x*-axis shows the number of amino acids. (**B**) Phylogenetic analysis of the amino acid sequences of ppci1. Homologous protein sequence sources were obtained from NCBI the Basic Local Alignment Search Tool, and the numbers on the branches represent the bootstrap values for 1000 replicates. (**C**) Comparison of the amino acids of ppci1 from other known fungi. Identical residues are shown in black color.

To identify orthologs of *Neurospora crassa ppci1* (XP_011393912) in *A. flavus*, the protein sequences of ppci1 (XP_011393912) from *N. crassa* was used as queries for Blast analyses in the using NCBI the Basic Local Alignment Search Tool (http://blast.ncbi.nlm.nih.gov/Blast.cgi). AFLA_050760 was predicted to primary structure analysis, the ppci1 protein contains 122 amino acids with 25 positively (Lys + Arg) and 18 negatively (Asp + Glu) charged residues. The predicted molar mass of ppci1 was 13,295 Da with theoretical pI of 9.49, and the grand average of hydropathicity (GRAVY) was −0.326. The whole protein contains about 18% alpha helix, which makes the protein’s overall structure to be much stable (Figure 1A). The phylogenetic analysis of ppci1 protein sequences from Aspergillus and other fungal species indicate that these proteins belong to the same family jointly in a single clade (Figure 1B). Furthermore, protein sequence of various fungi were downloaded, such as *A. flavus* (XP_002383203.1), *Endocarpon pusillum* (XP_007802438.1), *Cladophialophora bantiana* (XP_016623964.1), *Paracoccidioides brasiliensis* (XP_010757450.1), *A. taichungensis* (PLN84419.1), *Emmonsiacrescens* (KKZ58467.1), *A. clavatus* (XP_001269619.1), *A. nomius* (XP_015404128.1) (Figure 1C). The Alignment was done using DNAMAN software (http://www.lynnon.com) trial version 7.0.2.176.

### 2.2. Protein Expression and Purification

**Figure 2:**
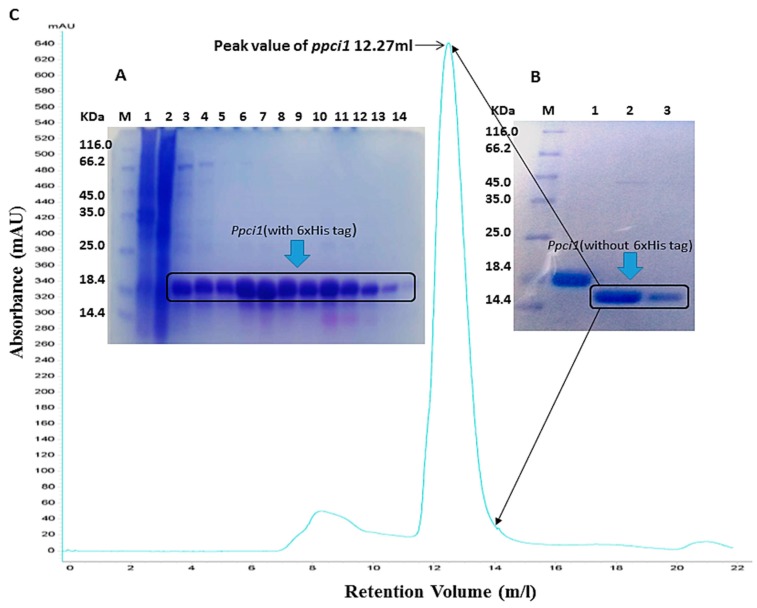
Expression and purification of ppci1. (**A**) Purification of ppci1 with 6×His tag from cell extract using Ni-NTA column chromatography. M: marker, Lane 1: supernatant after cell sonicated and centrifuged, Lane 2: flow-through fraction from Ni-NTA column, Lane 3–4: wash/elution fractions with 20 mM imidazole, Lane 5–11, elution fractions with 50 mM imidazole, Lane 12–13, elution fractions with 100 mM imidazole. Lane 14, elution fractions with 300 mM imidazole. (**B**) Purification of untagged ppci1 using a Ni-NTA column after the PreScission protease proteolytic reaction. M: marker, Lane 1: *ppci1* protein with 6 ×His tag (control), Lane 2–3 flow-through fractions from Ni-NTA column (20 mM imidazole). (**C**) Size exclusion chromatography analysis of untagged ppci1 by using a Superdex 75 10/300 GL column.

The expression vector pRSFDuet-1 was used and expressed product of *ppci1* in *E. coli* system was at high-level with excellent solubility. Then purification of 6×His-tagged ppci1 was removed by using a Ni-NTA column (Figure 2A). Furthermore, PreScission protease was used for removal of the 6×His-tag of the recombinant protein. About ninety percent of His-tag from ppci1 protein was removed even in the high-salt buffer, which contained 500 mM NaCl and 300 mM Imidazole. After proteolytic digestion, the untagged and tagged species were separated with a second Ni-NTA column by collecting the flow through fraction and the fraction with low concentration imidazole elution (Figure 2B). The untagged protein was further purified by a size exclusion chromatography column (SUPERDEX 75 10/300 GL, GE Healthcare Life Sciences), and the result showed that ppci1 was eluted at 12.27 mL with a single peak (Figure 2C). Finally, about 50 mg recombinant protein (>95% pure) was purified from 1 L cell culture.

### 2.3. Peptides Identification by Mass Spectrometry

**Figure 3:**
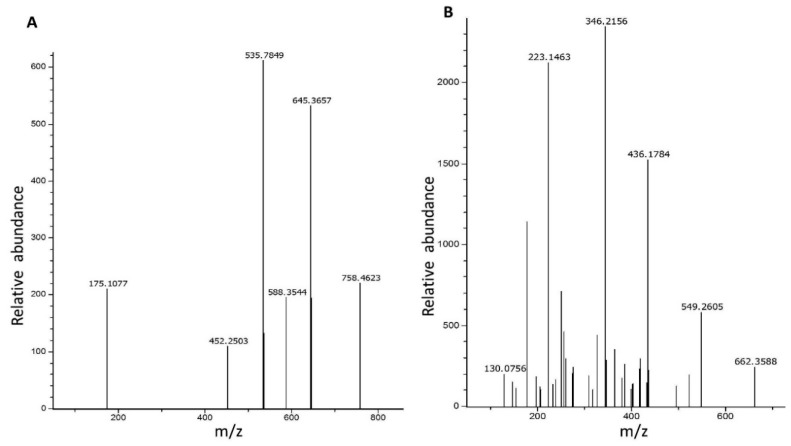
Determination of ppci1 peptide via mass spectrometry. (**A**) Annotation analysis of MS-MS spectrum of the peptide QGGSLGWKVR. (**B**) Determination of MS-MS spectrum of the peptide HILCEK. m/z = Mass to charge ratio.

The purified recombinant ppci1 protein was used for proteomic analysis via liquid chromatography-mass spectrometry. Total of five peptides QGGSLGWK, HILCEK, EFSEDKAR, QGGSLGWKVR, QGGSLGWKVR and two peptides HILCEK and QGGSLGWKVR (Figure 3) showed single domain polypeptides. A total of 1015 peptides were matched, and 16 non-duplicates and 999 duplicates were observed. Molar mass of target protein was 13,344 Da, and the score was 16,748 emPAI (Exponentially modified protein abundance index) 61.90 dare. These peptides matched the original sequence from NCBI, indicating the correct protein identification.

### 2.4. Substrate Specificity and Effect of Inhibitor On ppci1

**Figure 4:**
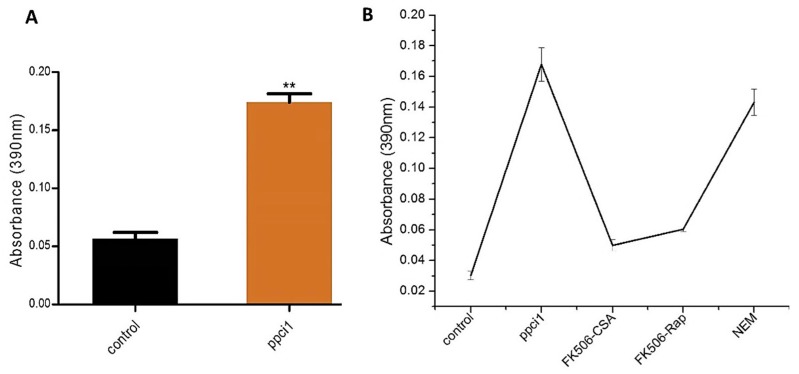
Activity of purified ppci1 and inhibition effect. (**A**) Activity of the ppci1 with the substrate compared with control (in the absence of ppci1). The asterisks ** represents a significant difference level of *p* < 0.001. (**B**) Effect of inhibitors (FK506-CSA, FK506-Rap and NEM).

The determination of PPIase activity was analyzed by proteolytic cleavage assay. The purified protein ppci1 activity was assayed at 15 °C in chymotrypsin in assay buffer (HEPES pH.8.0). Enzymatic activity was measured using absorption at 390 nm as shown in (Figure 4A), indicating that PPIase activity of the purified ppci1 protein increased with the substrate compared to control. Enzymatic activity of ppci1 was further investigated, based on their sensitivity to the immunosuppressive drugs, Tacrolimus (FK506) Cyclosporin A (CsA), Rapamycin (Rap). The result showed that, activity of ppci1 was inhibited by these aforementioned drugs with constantly decreased absorption (Figure 4B). ppci1 was also treated with NEM (*N*-ethylmaleimide) and the result indicated that NEM inhibited ppci1, but the effect was not as much obvious as inhibition result described above.

### 2.5. 3D Model of Ppci1

**Figure 5:**
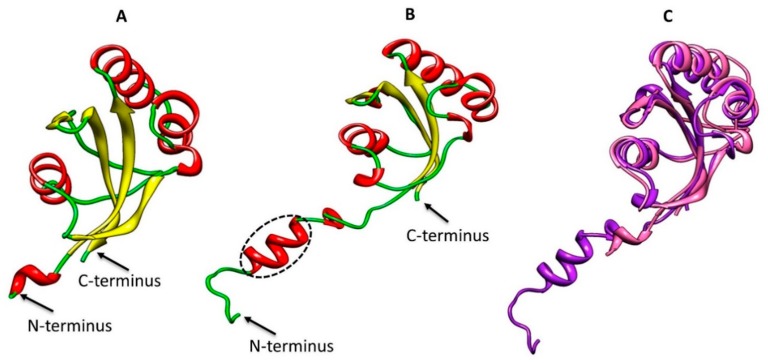
Structure prediction of ppci1 based on template (**A**) 3D structure of the template, red stands for alpha helices, yellow for sheets, and green for loops. (**B**) 3D predicted structure of ppci1. Predicted model contains one extra α-helix near the N-terminus (black circle). (**C**) Superposition model of ppci1: template (indigo) model (purple).

Prediction of three-dimensional protein structure was applied to characterize the structure and function of PPIase. To determine the sequence similarity between the template and target protein NCBI accession (No. XP-002383203). Crystallographic structure (PDB: 3ui6) was used as a template model to predict the structure of ppci1. Both models of template and target were shown in (Figure 5A,B). The finest predicted model was used for further analysis by PROCHECK [29], and this model also tests φ and ψ torsion angles by using a Ramachandran plot. Analysis of the Ramachandran plot showed that 93.8% residues of the main chain were within the most favored or allowed region and 6.2% residues were in the additionally allowed region. The Root Mean Square Deviation (RMSD) between these two structures was 1.0Å, and the low RMSD also indicated the strong structural homology between the two models. The value of Z-scores from the obtained model was −7.16, which was remarkably closed to the value of template one (−7.88). It indicates that the predicted model was consistent with previous X-ray structure, and the overall predicted model was generally similar to the template. The major difference between template and the predicted model was found at the N-terminus (Figure 5B). There were structural variances observed in the loop regions with some structural flexibility or missing residues (Figure 5C).

### 2.6. Construction of Δppci1 Deletion Mutant 

**Figure 6:**
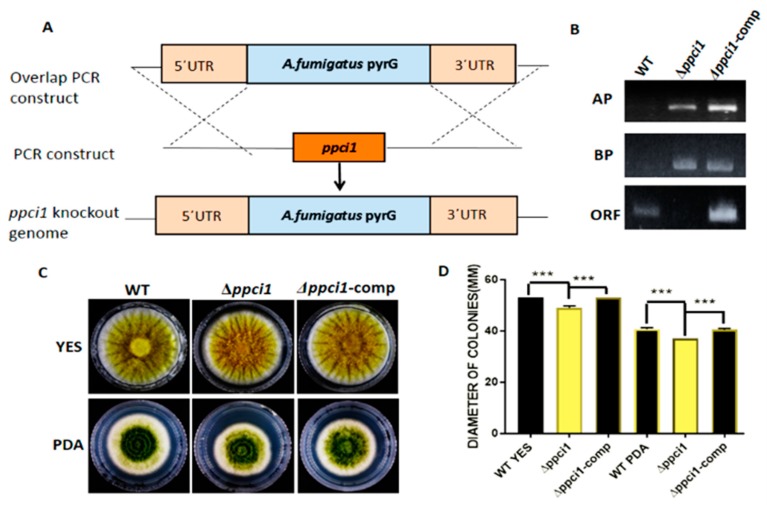
Construction of *ppci1* deleted Δ*ppci1*, complemented Δ*ppci1*-comp strains and growth analysis. (**A**) Schematic diagram of gene deletion method. The fragments 5′UTR (Untranslated region) and 3′UTR were amplified with primers fused together AF/AR, BF/BR, *pyrG*-F/*pyrG*-R. (**B**) PCR confirmation of Δ*ppci1* and Δ*ppci1*-comp strains with genomic DNA as a template (ORF) open reading frame, AP and BP fragments. (**C**) Phenotype analysis of WT, Δ*ppci1* and Δ*ppci1*-comp strain grown PDA and YES medium at 37 °C for 5 d in the dark. (**D**) The asterisks ***means a significant difference (*p* < 0.01).

In order to study the function of *ppci1* in *A. flavus*, we generated *ppci1* deletion mutant (Δ*ppci1*), and its complementation strains (Δ*ppci1*-Comp). The *ppci1* gene was knocked out by homologous recombination (Figure 6A). The screening gene *pyrG* of *A. fumigatus* was used for target gene deletion. The complementation strain Δ*ppci1*-Comp with a wild-type copy of *A. flavus ppci1* was constructed using the pyrithiamine antibiotic marker (*ptrA*). Furthermore, selected transformants were confirmed to be knockouts or complementation strains by PCR (Figure 6B). To study the effect of *ppci1* gene deletion on the growth of *A. flavus*, the wild-type (WT), Δ*ppci1 and* Δ*ppci1-Comp* strains were inoculated on PDA and YES medium. Moreover, diameters were measured under the same growth conditions of *A. flavus* (Figure 6C/D). The results demonstrated that Δ*ppci1* displayed significantly decreased radial growth compared to the WT and Δ*ppci1-Comp* strains.

### 2.7. Effect of Inhibitors on the Growth of Fungal Strain

**Figure 7:**
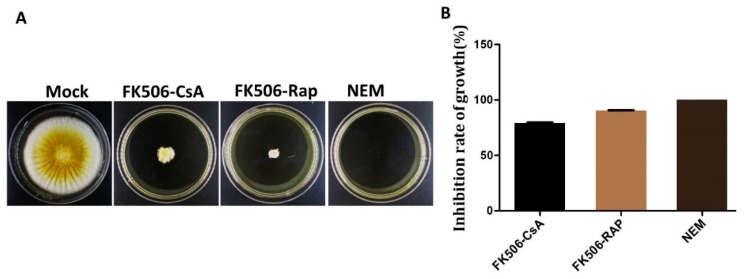
Effect of different inhibitors on the fungal growth. (**A**) Phenotype analysis of WT strain with the inhibitors Fk506-CsA, Fk506-Rapamycin and NEM at 37 °C for 5 d in the dark. (**B**) Inhibition rate of growth analysis with different drugs.

As stated earlier, *A. flavus* is a pathogen. It is necessary to decrease its pathogenic ability to prevent infections. In this study, we treated *A. flavus* wild-type strain with FK506-CsA, FK506-Rap and NEM inhibitors. Whereas, untreated strain was used as a control (mock). Every strain was then inoculated into YES medium, and cultured at 37 °C in the dark for 5 d. The inhibition growth rate was measured by diameter and results showed that the strains treated were significantly decrease in the growth rate by inhibitors. Interestingly, the strain treated with NEM inhibitor, completely inhibited fungal growth (Figure 7A,B).

### 2.8. Ppci1 is Important for Conidiation and Sclerotia Formation in A. flavus

**Figure 8:**
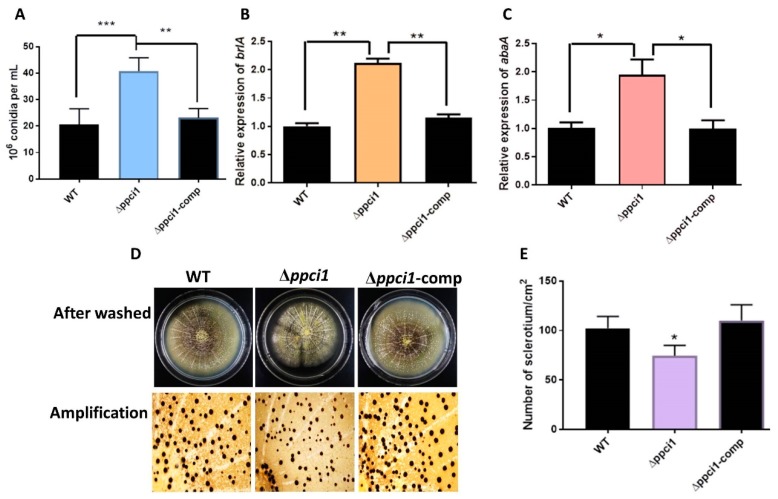
Conidiation and sclerotial phenotype of WT, Δ*ppci1* and Δ*ppci1*-comp strains. **(A)** Observation of conidiation WT, Δ*ppci1* and Δ*ppci1-Comp* strains. (**B**) Analysis of reverse transcription polymerase chain reaction (qRT-PCR) on the expression level of the target gene. (**C**) Analysis of *abaA* gene related to conidia formation by qRT-PCR, used to evaluate the target gene expression level. (**D**) Sclerotial phenotype of WT, Δ*ppci1* and Δ*ppci1-Comp* strains cultured on the WKM medium after ethanol treatment. Enlarged image of the plate was provided downside. (**E**) Quantitative analysis of sclerotia production. *, ** and *** indicate significance levels of *p* < 0.05, *p* < 0.01 and *p* < 0.001, respectively.

To analyze the bio-function of *ppci1* gene in conidiation, the strains (WT, Δ*ppci1*, and *ppci1*-Comp) were inoculated into YES medium, and cultured at 37 °C in the dark for 5 days. A quantitative analysis showed that Δ*ppci1* produced a large number of conidiophores compared to WT and *ppci1*-Comp strains (Figure 8A). Moreover, a quantitative qRT-PCR was used to analysis the expression level of *brlA* and *abaA*, which were two conidia related transcriptional factor encoding genes and influence conidial formation [30]. The expression level of *brlA* was higher than *abaA* in the Δ*ppci1* mutant compared with WT and *ppci1*–Comp strains (Figure 8B,C). In order to examine the effect of *ppci1* on sclerotia formation in *A. flavus*, Wickerham (WKM) medium was inoculated with the all strains and cultured for 7 d at 37 °C in the dark. Then 70% ethanol was used to wash off aerial hyphae and conidia to visualize sclerotia, and the result demonstrated that the Δ*ppci1* mutant displayed a significant reduction of sclerotia compared to WT and *ppci1*–Comp strains (Figure 8D,E). All these results demonstrated that *ppci1* was involved in conidiation and sclerotia formation in *A. flavus*.

### 2.9. Effects of ppci1 on Aflatoxin Biosy$nthesis and Pathogenicity

**Figure 9:**
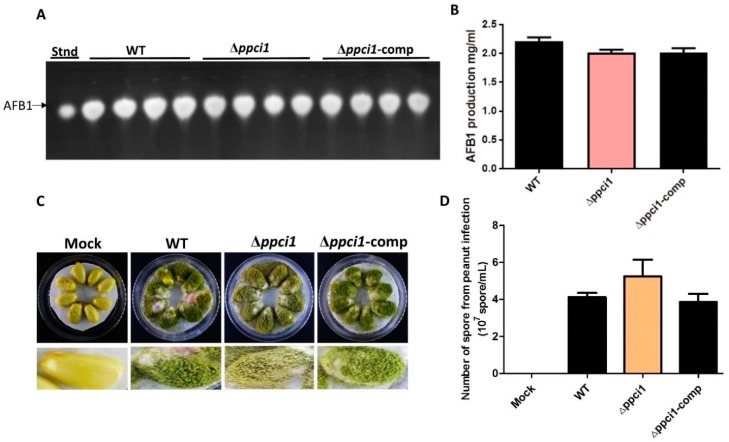
Aflatoxin production and seed infection of WT, Δ*ppci1* and Δppci1-comp of *Aspergillus flavus* (**A**) Aflatoxin B1 production was analyzed by TLC (Thin layer chromatography) in the indicated strains. (**B**) Quantitative analysis of AFB1 as shown in (A). (**C**) Peanut cotyledons were infected by WT, Δ*ppci1* and Δ*ppci1*-comp strains. (**D**) Quantitative analysis of spores collected from peanut cotyledons.

*A. flavus* is virulent, on the basis of aflatoxin production. To examine the effect of *ppci1* on aflatoxin production, all the strains (WT, Δ*ppci1*, and *ppci1*-Comp) incubated in YES liquid medium at 29 °C in the dark. After 5 d incubation, mycotoxins were extracted and analyzed on TLC plate, which showed a slight decrease of aflatoxin production in Δ*ppci1* strain compared to that of WT strain (Figure 9A,B). Furthermore, to examine the effect of *ppci1* on pathogenicity in *A. flavus*, the WT, Δ*ppci1* and *ppci1*–Comp strains were inoculated with peanut seeds. After 5 d of inoculation, spore productions in the infected seeds were examined. The deletion of *ppci1* resulted in increased spore production compared to WT and Δ*ppci1-Comp* strains (Figure 9C,D). All these results demonstrated that *ppci1* was not involved in Aflatoxin biosynthesis but might have an influence on pathogenicity to crop seeds in *A. flavus*.

## 3. Discussion

The Peptidyl Prolyl *cis-trans* Isomerases (PPIases) are highly conserved proteins that originate in eukaryotic and prokaryotic cells, which based on the drug specificity and primary sequence homology [31,32]. It has been reported that, PPIases play critical roles in cell process such as protein secretion, RNA processing and cell cycle regulation to pathogenicity [33,34]. However, to know whether the coding gene of PPIases plays a role in *A. flavus*, we first identified the gene of PPIases from *A. flavus* named *ppci1*. The protein sequence belongs to rotamase-2 super family, which contains conserved domain FKBP like Immunophilins. The *ppci1* gene was cloned into expression vector. The over expression of the chimeric plasmid in a bacterial host produces large quantities of the encoded protein [35]. Ni-NTA affinity chromatography was effective, and the one-step method was used to separate target proteins with His-tags using this method. Thus, we were capable of obtaining pure ppci1-His fusion protein (Figure 2). Our results were almost the same as a previous study such as the purity of nucleoside diphosphate kinase proteins, which were shown can reach >98% [36]. We further performed mass spectrometry analysis to get the molar mass and insights into different peptides. In the previous study, a novel PPIase from *E. coli* was determined by mass spectrometry [37]. Our results also showed the PPIase activity of purified protein (Figure 4). In the previous study, the purified recombinant fusion protein AtCyp19–3 showed PPIase activity [38]. A previous study had identified that the activity of recombinant PPIase from wheat was inhibited by rapamycin and FK506 [39]. The enzymatic activity of AtCyp19–3, was specifically inhibited by CsA [38]. Primary targets of PPIases were immunosuppressive drugs CsA, rap and FK506 [40]. In this study, we used all the above inhibitors which inhibited the activity of purified ppci1 protein but FK506, CsA significantly inhibited the activity.

To examine the in vivo function of *ppci1* in *A. flavus*, Δ*ppci1* and Δ*ppci1-Comp* mutant strains were constructed. Δ*ppci1* strain showed less growth in dark compared with WT and Δ*ppci1-Comp* strains. An alteration in sclerotia numbers were found in the Δ*ppci1* strain, which results in decreased sclerotia in the mutants. As the previous study of gene acyA from *A. flavus*, the deletion mutant strain blocked the sclerotia production [41]. These results indicated that *ppci1* may play an important role in regulating sexual development in *A. flavus*. Here, we found that the conidia growth rate of the Δ*ppci1* mutant was significantly increased (Figure 8), which was alike to the previous study of gene *NmrA* from *A. flavus*, deletion mutant increased production of conidia [42]. *A. flavus* produces a sexual spores (conidia), and the *ppci1* deletion resulted in significant increase in conidiation production compared with the WT and complementary strains. These results indicated that *ppci1* may play a critical role in regulating asexual development in *A. flavus*. It was reported that, *A. flavus* produces aflatoxin B1 and B2 [43], which were biosynthesized through a highly refined pathway, and it could be affected by numerous biotic and abiotic factors [42]. This study demonstrated that the aflatoxin produced by Δ*ppci1* was slightly less than that of the WT strain. This finding is closely similar to the study of gene *PbsB*, deletion mutant produce aflatoxin [44]. *A. flavus* on peanut seeds, resulting in high colonization as reflected by large spore production [45]. To investigate the bio-function of *ppci1* in *A. flavus* pathogenicity, we observed seeds infection in the Δ*ppci1* mutant, and the result indicated that, it led to increased colonization on peanut seed (Figure 9C). This showed that *ppci1* could also be involved in virulence and pathogenicity. For the analysis of sensitivity of *A. flavus* to the inhibitors, Rap, CsA, FK506 and NEM were used to treat an *A. flavus* WT strain. The results showed that growth of *A. flavus* was highly sensitive to both FK506-CsA and FK506–Rap whereas under NEM, growth was totally inhibited. In the previous study on gene regulator *nmrA* in *A. flavus*, the Rap was used to inhibit the growth of WT and mutant [42]. This result demonstrated that all the above inhibitors play a key role in decreasing the growth of *A. flavus*.

## 4. Materials and Methods

### 4.1. DNA Cloning, Protein Expression and Purification

RNA from *A. flavus* NRRL3357 (stored in our lab) was isolated by using the Total RNA Extraction Kit. The cDNA for the *ppci1* gene was synthesized from RNA using the Revert Aid First Strand cDNA Synthesis Kit. Then the gene was amplified by PCR and cloned into the modified pRSFDuet-1 expression vector. The plasmid for expression contains a 6 × His tag followed by a PreScission protease cleavage position at the N-terminus of the target protein. The plasmid containing the target gene was constructed and transferred into *E. coli* BL21 (DE3) competent cells. After that, transformed cells were cultured in 10 mL LB medium containing 50 mg/L kanamycin overnight at 37 °C. Then cell culture was inoculated into 1L LB medium at 37 °C till the OD 600 nm optical density reached 0.5, then induced at 16 °C overnight with 0.3 mM isopropyl-ß-D-thiogalactopyranoside (IPTG). Finally, the cells were centrifuged at 6000 *g* for 5 min and stored at 4 °C. Details regarding primer, plasmid, host, ppci1 protein sequence and all Buffers are shown (Table 1). The cell pellet was washed with binding buffer A and then sonicated on ice. After that, cell lysate was centrifuged at 15,000 *g* for 20 min twice at 4 °C, and the supernatant was loaded onto Ni-NTA column. Then column was eluted with buffer A, B and C, respectively. The 6 × His- Peptidyl-prolyl *cis-trans* isomerase fusion protein was eluted in buffer A, B, and C fraction. To remove the 6 × His tag, the fusion protein was incubated with Buffer C and PreScission protease at 4 °C for 16 h [36]. After the digestion reaction, the mixture was dialyzed against buffer D, and then loaded onto another Ni-NTA column (All Buffers are shown in Table 1). The untagged protein of *ppci1* was collected, and further purified by size exclusion chromatography (Superdex 75 10/300 GL).

### 4.2. Mass Spectrometry

Mass spectrometry analysis of ppci1 was performed by Beijing Protein Innovation. The band containing ppci1 was excised from the SDS-PAGE (Sodium dodecyl sulfate polyacrylamide gel electrophoresis) then digested with a reagent containing 50% acetonitrile and 25 mM ammonium bicarbonate. Complete absorption of colloidal particles was carried out with 10 mM DTT (dithiothreitol) by incubating at 50 °C for 60 min. Furthermore, DTT was removed, and 55mM IAM (Iodine acetamide), was added then incubated at room temperature for 45 min. After the removal of excess IAM, the sample was washed with 25 mM ammonium bicarbonate for 10 min, twice. The enzyme was diluted with 25 mM ammonium hydrogen carbonate and added into the dehydrated colloidal particles for full absorption. Finally, digestion reaction was terminated with 0.1% concentrated FA (formic acid), and the sample was detected via Q-TOF (Quadrupole Time-of-Flight). Protein identification was performed with the Mascot search algorithms [46].

### 4.3. PPIase Activity Assay

PPIase activity was assayed for 360s at 15 °C in a coupled reaction through chymotrypsin. The assay mixture 1 mL, contained 80 μL succinyl-ala-ala-prophe-p-nitroanilidine (test peptide), and purified proteins 25 µL with assay buffer (HEPES 50 mM pH 8.0, 150 mM Triton X-100, NaCl 0.05%). The absorbance change was observed at 390nm by spectrophotometer [38], and the inhibition effects of Rapamycin (Rap), Tacrolimus (FK506) and cyclosporine A (CsA) were examined. After that final concentration slightly modified of FK506, CsA and Rap were 0.160–0.65 µL, 0.050–1.40 µL and 0.055–1.60 µL. Rap or FK506 was added into the assay mixture containing the purified ppci1 (25 µL), respectively and analyzed by spectrophotometer. Then the mixture was incubated with ppci1, followed by dilution in assay buffer with 500 µM NEM (*N*-ethylmaleimide) from 50 mg/mL stock made in 100% EtOH) at room temperature. PPIase activity as described above [47]. Software Origin 6.0 and Graph Pad Prism 5 was applied in the analysis of the obtained data.

### 4.4. Reconstruction of Phylogeny Based on Sequence

All available PPIase sequences were collated by querying NCBI (National Centre of Biotechnology Information), protein database. Multiple sequence alignment was constructed by DNAMAN software (http://www.lynnon.com) using trial version 7.0.2.176. was utilized to generate the phylogenetic tree, by using the Neighbor-Joining method with Poisson distribution, pairwise deletion, and bootstrap values of 1000 replications [48].

### 4.5. Structure Determination and Functional Analysis

The primary structure analysis of *ppci1* was predicted by using Expasy’s Portramservere. For determination of secondary structure, SOPMA was applied. The functional sites were recognized by using a ScanProsite tool in Expasy. Model comparing was initiated by reorganization of PDB protein structures via the use of query sequence as a target [49]. The target sequence matched with the sequence of each structure in a database [50]. PDB viewer was used to generate a structure-based alignment, and SWISS-MODEL was utilized in the optimization model to minimize energy. ProSA, SAVES and ERRAT programs were used for the validation of model [51]. The RMSD value between the model structure and template was calculated using SPDBV program.

### 4.6. Gene Deletion and Complementation of Ppci1

To generate the deletion strain (Δ*ppci1*), an 873ap fragment upstream from *ppci1* was amplified with *ppci1*AP1/FR and *ppci1*BP1/FR primers. Then, a 948bp fragment downstream from *ppci1* was amplified with *ppci1*A/P and *ppci1*B/P primers. The total DNA extracted from *A. flavus* was used as a template. To generate the fragment containing the upstream fragment, the *pyrG* selectable marker as well as downstream fragments were added sequentially. A fusion PCR product was used to generate the *ppci1* mutants, and the product was transformed into protoplasts of the wild-type CA14. Protoplast preparation and transformation were done following the as previous protocols [52]. The primers, used in this study are shown in (Table 2). For complementation, the *ppci1* coding region and promoter region was amplified using primers *ppci1*-comp-F/R from the genomic DNA from the *A. flavus* wild-type (WT) strain, after that cloned into the digested pPTRI vector by T4 DNA ligase (Takara). The recombinant pPTR-*ppci1* was transformed into protoplasts of the Δ*ppci1* mutant with pyrithiamine selectable marker. Protoplast preparation was performed as previous protocol [53], and the mutants were verified by PCR.

### 4.7. Physiological Growth, Sclerotia and Conidiation, Analysis

The phenotypes strains (WT, Δ*ppci1*, and Δ*ppci1*-Com) were inoculated in PDA (Potato dextrose agar) and YES (Yeast extract supplement) medium. For the purpose of colony morphology and mycelial growth, all the strains were cultured at 37 °C in the dark. After 5 d colony diameters were measured. For conidia analysis, all strains were inoculated and cultured on YES medium at 37 °C in the dark. After 2 d, the hyphae were cut and observed under a microscope [44]. The qRT-PCR was used with the Real-Time PCR system (Thermo Scientific, Finland) and SYBR Green Premix kit (Takara, Dalian, China). The 2^−ΔΔCT^ method was used to evaluate the expression level of the target gene [30]. For sclerotial analysis, all strains were inoculated and cultured on WKM (Wickerham) agar medium at 37 °C in the dark. After 7 d, conidia on the surface of the medium were washed away by 75% ethanol, and the sclerotia was examined under the microscope [54].

### 4.8. Effect of Inhibitors

To determine the role of the *ppci1* gene in *A. flavus* response to inhibitors, all strains were inoculated into YES Medium. Then inhibitors FK506-CsA, FK506-Rap and NEM were added into different WT strain then each one inoculated into petri dish containing 15mL of YES medium. The cultures were then incubated for 5 d at 37 °C in the dark. The diameter of the colony was measured and relative inhibition rates was calculated by using this formula: Inhibition rate of growth = (diameter of Mock strain - diameter of inhibited strain)/(diameter of Mock strain) × 100.

### 4.9. Aflatoxin and Seeds Infection Assays

The AFs were extracted and analyzed by TLC (Thin layer chromatography) in a solvent system (chloroform: acetone = 9:1), then examined under UV light at 365 nm. For the measure of quantitative analysis of the AF production, Gene Tools software was used [5]. To test the ability of the Δ*ppci1* mutants to infect crop seeds, the peanut cotyledons were inoculated with the same concentration of spore from WT and mutants of *A. flavus*. After incubation for 6 d, the peanuts were harvested in 50 mL Falcon tubes, and then vortexed for 5 min to release the spores into 15 mL of sterile water. Spores number was counted under the microscope [42]. All the data were analyzed by Graph Pad Prism 5 software.

## 5. Conclusions

This was the first report on the functional study of PPIase in *A. flavus.* The gene of *ppci1* was cloned from *A. flavus* and expressed as recombinant protein in an *E. coli* system. The yield of recombinant protein without a His tag was > 95% pure. The purified ppci1 protein was characterized by mass spectrometry, and identified peptides were matched with the original sequence from NCBI (National Center for Biotechnology Information), indicating the correct protein identification. The ppci1 activity was measured with the substrate and treated with different inhibitors. We found that FK506, CsA and Rapamycin inhibit the protein activity of ppci1. A 3D protein model of ppci1 was built based on its primary sequence. Furthermore, the homologous recombination method was used to construct the *ppci1* gene deletion strain. The deletion mutant Δ*ppci1* showed a decrease in growth and sclerotia production but increased in conidiation when compared with WT and Δ*ppci1-Comp*, and caused pathogenicity on peanut seeds. The inhibitors were used to treat the fungal strains and we found that, inhibitors significantly inhibited growth rates. Our study provides new insights into the role of *ppci1* in *A. flavus* on the basis of prevention and control of *A. flavus* pathogenicity in the earlier stages, which could be taken as crucial targets for designing the antifungal drugs.

## Figures and Tables

**Table 1:** The production macro molecules information of *ppci1* Protein.

Source Organism	*A. flavus NRRL3357*
Primer F	5′-ATGGCGCCCAAAAACA3′
Primer R	5′-TTACTTCCGCCCCTCGA3′
Cloning and Expression Vector	pRSFDuet-1 expression vector (Novagen)
Expression Host	*E. coli* BL21(DE3)
Complete amino acids sequence of the constructed product	MAPKNKGGDKKGKGNDGGDKGGKGLKPATSINVRHILCEKHSKKEEALEKLRNGSKFDDVAREFSEDKARQGGSLGWKVRGSLDGTFEKAAYELEPSTTANPKYVEVKTGFGYHIIMVEGRK
Binding Buffer A	50 mM Tris-HCl, 500 mM NaCl, 20 mM Imidazole pH 8.0
Eluted Buffer A	50 mM Tris-HCl, 500 mM NaCl, 20 mM Imidazole pH 8.0
Eluted Buffer B	50 mM Tris-HCl, 500 mM NaCl, 50 mM Imidazole pH 8.0
Eluted Buffer C	50 mM Tris-HCl, 500 mM NaCl, 300 mM Imidazole pH 8.0
Dialyzed Buffer D	50 mM Tris-HCl, 500 mM NaCl

**Table 2:** Specific primers used for PCR.

Primers	Sequence (5′–3′)
*ppci1* AF	CCTAGCGACTCAAAGCG
*PPci1* AR	GGGTGAAGAGCATTGTTTGAGGCTTGGGTAACGGTAAGTGC
*ppci1* ORF/F:	AACAAAGGCGGAGACAA
*ppci1* ORF/R:	AAGGAAAGGAGACGAAAG
*ppci1* BF	GCATCAGTGCCTCCTCTCAGACGCATTACTTTACTGGCTCTT
*ppci1* BR	GTCTACATTTGCCGCTAT
pyrg F:	GCCTCAAACAATGCTCTTCACCC
pyrg R	GTCTGAGAGGAGGCACTGATGC
Comp F:	ACAAGCGTTCCAAGCCA
Comp R:	TTCCGCCCCTCGACCAT
